# ITGB1b-Deficient Rare Minnows Delay Grass Carp Reovirus (GCRV) Entry and Attenuate GCRV-Triggered Apoptosis

**DOI:** 10.3390/ijms19103175

**Published:** 2018-10-15

**Authors:** Geng Chen, Lv Xiong, Yumeng Wang, Libo He, Rong Huang, Lanjie Liao, Zuoyan Zhu, Yaping Wang

**Affiliations:** 1 State Key Laboratory of Freshwater Ecology and Biotechnology, Institute of Hydrobiology, Chinese Academy of Sciences, Wuhan 430074, China; chengeng@ihb.ac.cn (G.C.); 18963973548@163.com (L.X.); wymlemon@whu.edu.cn (Y.W.); helibowudi@ihb.ac.cn (L.H.); huangrong@ihb.ac.cn (R.H.); liaolj@ihb.ac.cn (L.L.); zyzhu@ihb.ac.cn (Z.Z.); 2 University of Chinese Academy of Sciences, Beijing 101408, China; 3 College of Life Sciences, Wuhan University, Wuhan 430072, China

**Keywords:** grass carp, grass carp reovirus, integrin, endocytosis, clathrin, apoptosis

## Abstract

Integrin β-1 (ITGB1) is a transmembrane protein belonging to the integrin family and it plays an important role in viral entry. In this study, the *itgb1b* gene of the rare minnow, *Gobiocypris rarus*, was cloned and analyzed. To investigate the possible role of *itgb1b* on grass carp reovirus (GCRV) infection, we generated an ITGB1b-deficient rare minnow (ITGB1b^−/−^) using the CRISPR/Cas9 system. Following stimulation with GCRV, the survival time of the -ITGB1b^−/−^ rare minnows was extended in comparison to the wild-type minnows. Moreover, the relative copy number of GCRV and the level of clathrin-mediated endocytosis-associated and apoptosis-related gene expression in the ITGB1b^−/−^ rare minnows was significantly lower than that of the wild-type minnows. These results suggested that the absence of *itgb1b* reduced viral entry efficiency and the expression of apoptosis-related genes. Moreover, the data suggested that *itgb1b* played an important role in mediating the entry of viruses into the cells via clathrin. Therefore, these findings provide novel insight into the function of *itgb1b* in the process of GCRV infection.

## 1. Introduction

Grass carp, *Ctenopharyngodon idella*, is one of the most important aquaculture species in the world, accounting for 13% of global freshwater aquaculture production in 2015 [1,2]. However, grass carp haemorrhage disease, caused by the grass carp reovirus (GCRV), is one of the most damaging diseases, resulting in huge economic losses to the grass carp aquaculture industry. GCRV was first isolated in China and belongs to the genus *Aquareovirus* of the family *Reoviridae* [3]. Moreover, GCRV is a double-stranded RNA virus and may trigger apoptosis in grass carp kidney cells [4]. Understanding the mechanism by which GCRV enters cells and induces apoptosis is critical for developing virus-resistant strains of grass carp. Rare minnow, *Gobiocypris rarus*, is a Chinese native species belonging to the family *Cyprinidae,* which can be infected with GCRV, resulting in mortality as high as 100% [5]. Therefore, due to its biological characteristics, rare minnow has the potential to be a model for aquatic toxicity testing, chemical safety assessments, and antiviral breeding [6].

Integrin β-1 (ITGB1) is a member of the integrin family that comprises a highly conserved heterodimeric transmembrane protein, which mediates adhesion to extracellular matrices and facilitates cell-to-cell contact and participates in many cell cycle processes as a guide molecule for signal transduction [7,8]. Integrins consist of two non-covalently bound alpha and beta glycoprotein subunits, and in mammals, a combination of 18 alpha and 8 beta subunits produces at least 24 different integrin dimers, which are substantially expressed on all cell types [9]. Moreover, integrins are used as receptors for several viruses, including adenovirus [10], foot-and-mouth disease virus [11], and Hantavirus [12].

In our previous studies, we found that there was an interaction between GCRV virions and the ITGB1 protein (data not published). In grass carp kidney cells, the most prominent pathway of differentially expressed genes enrichment during the early stages of GCRV infection is focal adhesion and extracellular matrix receptor interaction, where integrin plays an important role [4]. Furthermore, an analysis of ITGB1 expression during the embryonic development of zebrafish, showed that *itgb1* is involved in the formation of embryonic blood vessels and the heart [13].

Interestingly, among other species, *ITGB1* is often viewed as a potential therapeutic target for certain diseases. In the human lung adenocarcinoma cell line, SPC-A-1, *ITGB1* has been shown to play an important role in the development and metastasis of lung cancer [14]. In addition, targeting *ITGB1* with microRNA-124 can inhibit the adhesion and motility of oral squamous cell carcinoma (OSCC) [15]. In mouse experiments, it was found that miR-29c can act as a tumor suppressor in gastric cancer by directly targeting *ITGB1* [16]. Moreover, ITGB1^−/−^ mouse cells showed that the lack of *ITGB1* resulted in a reduction in West Nile virus (WNV) virions by more than 70% to 90% [17]. However, the understanding of the function of *itgb1* in teleost fish is limited, and the specific role of *itgb1* in the process of cell infection by teleost fish viruses remains unclear.

This study investigated the mechanism by which GCRV infects cells and the possibility of knocking out *itgb1b* to provide an innovative strategy for increasing the survival rate of the rare minnow following viral infection. In this study, the expression pattern of the rare minnow *itgb1b* gene in different tissues was cloned and analyzed, and the response to GCRV infection was studied. In addition, we also generated an ITGB1b^−/−^ rare minnow, in which both GCRV entry and virus-triggered apoptosis were inhibited compared to wild-type rare minnows. Our study elucidated the role of *itgb1b* in mediating endocytosis and provided new insights into the processes associated with GCRV-infected cells.

## 2. Results

### 2.1. Characterization and Phylogenetic Analysis of the ITGB1b Gene

Grass carp *itgb1b* (Genbank accession number: MG757434) has a full-length genome of 2352 bp and encodes a protein that is comprised of 783 amino acids. The rare minnow *itgb1b* (Genbank accession number: MG757435) is 2193 bp long and encodes a 730 amino acid predicted polypeptide.

**Figure 1:**
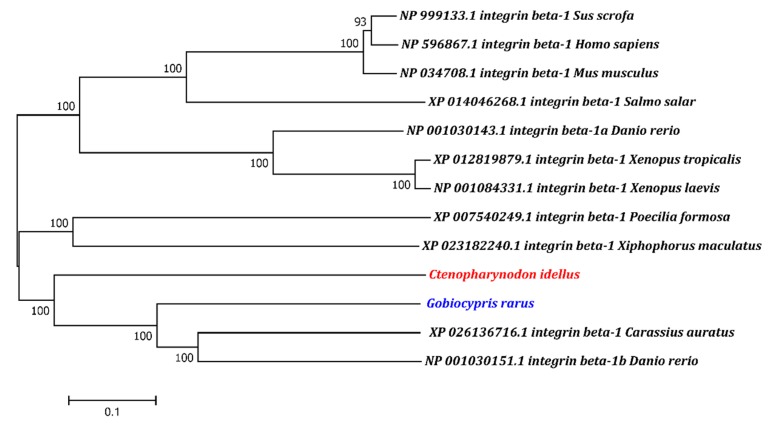
Phylogenetic relationship of the ITGB1 proteins in different species. A phylogenetic tree was constructed using the MEGA7 software. *Ctenopharynodon idellus* was highlighted in red and *Gobiocypris rarus* was in blue. *Sus scrofa*, *Homo sapiens*, and *Mus musculus* were introduced as outgroups.

To determine the evolutionary status of *itgb1b*, according to teleost fish (i.e., Ctenopharynodon idellus, Gobiocypris rarus, Danio rerio, Poecilia Formosa, Xiphophorus maculatus, and Salmo salar), amphibians (i.e., Xenopus laevis and Xenopus tropicalis) and mammals (i.e., Homo sapiens, Mus musculus, and Sus scrofa), a phylogenetic tree was constructed. As shown in Figure 1, the results show that *itgb1b* from teleost fish falls into one branch (except itgb1a from zebrafish), and ITGB1 from amphibians and mammals is considered to be an outer group.

### 2.2. Generation of an ITGB1b-Deficient Rare Minnow Using the CRISPR/Cas9 System

**Figure 2:**
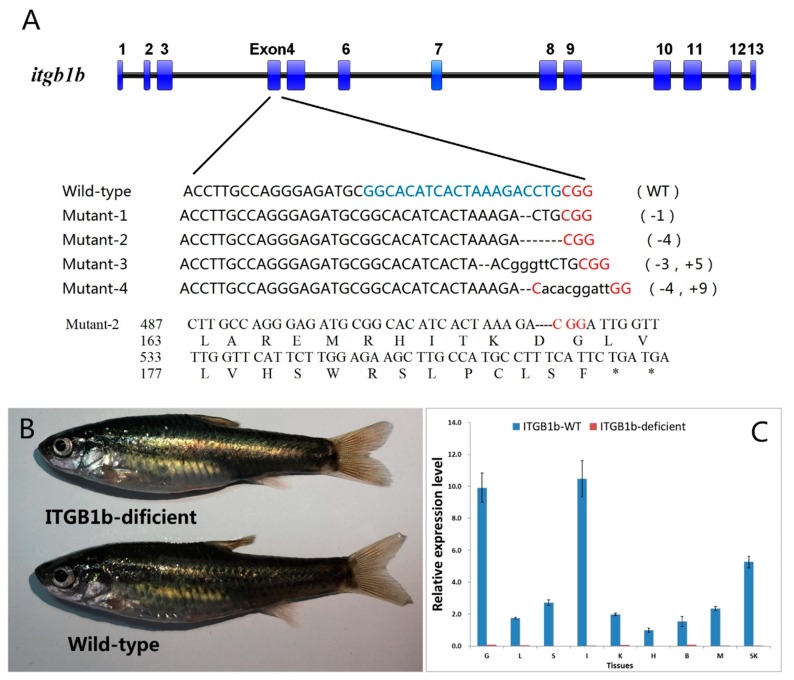
Mutations induced by the CRISPR/Cas9 system in the target sequence. (**A**) The thick, thin blue strips in the top shown as exons. Numbers to the right indicated the loss or insertion of bases for each allele. Deletions are shown as black dotted line and insertions as lower-case letters. The wild-type sequence is presented at the top, with the target site highlighted in blue and the PAM(protospacer adjacent motif) sequence highlighted as red. (**B**) Wild-type and ITGB1b-deficient rare minnows at 4-month-old. (**C**) Confirmation of loss of *itgb1b* expression level by RT-qPCR. β-actin was used as an internal control. The relative expression level was presented as the ratio of gene expression level in different tissues relative to that in the heart tissue from the wild-type fish. G, Gill; L, Liver; S, Spleen; I, Intestine; K, Kidney; H, Heart; B, Brain; M, Muscle; SK, Skin.

To further investigate the role of the *itgb1b* gene in fish, we chose the rare minnow as a model fish to generate an ITGB1b-deficient model via the CRISPR/Cas9 system. The target sequence (5′-GGCACATCACTAAAGACCTGCGG-3′) was located at 503 bp downstream of the translation start site (ATG) of the *itgb1b* gene in the rare minnow. Thus, mutations in the target sequence can result in a loss of function. Figure 2 shows the mutation introduced at the *itgb1b* target site (Figure 2A). A −4 bp mutation was selected for further study, which caused the 190th codon to be mutated into a termination codon. Wild-type and ITGB1b-deficient rare minnow at 4 months of ITGB1b-deficient rare minnow normal development, shows deformity does not appear (Figure 2B). In addition, RT-qPCR(RealTime QuantitativePCR) was performed to confirm that CRISPR/Cas9 induced a loss of function mutation in the rare minnow *itgb1b* gene. As shown in Figure 2C, no or a minimal expression level of *itgb1b* (Ct value ≥ 32) was detected in ITGB1b-deficient fish, whereas the expression level of *itgb1b* in wild-type was normal (Ct value range from 24~26).

### 2.3. ITGB1-Deficient Rare Minnow Exhibits Delayed Death Following GCRV Infection

**Figure 3:**
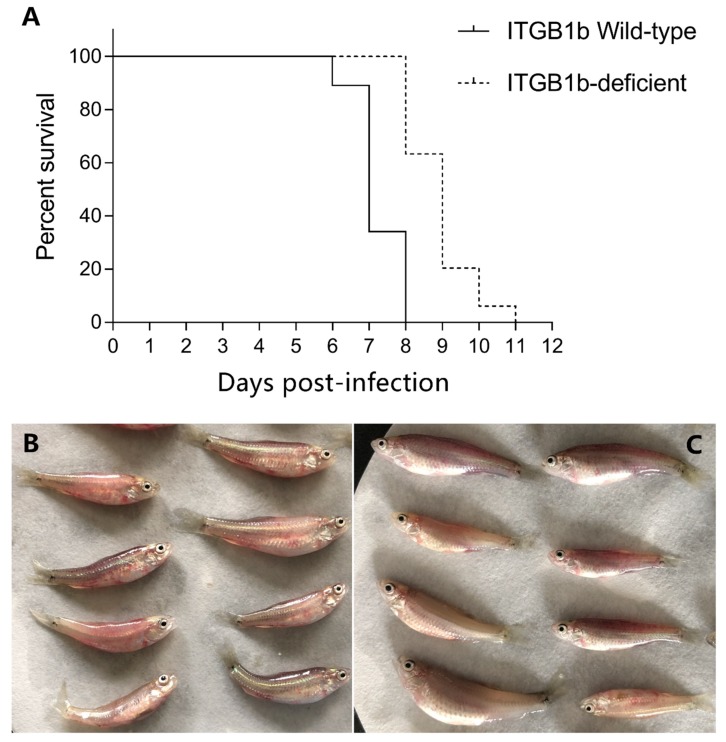
(**A**) Survival curve of ITGB1b-deficient and wild-type rare minnows after grass carp reovirus (GCRV) infection. ITGB1b-deficient and wild-type rare minnows were immersed into GCRV solution for 20 min and transferred to aerated freshwater and cultured at 28 °C. The dead fish in both groups were recorded every day. The clinical symptom of wild-type rare minnows (**B**) and ITGB1b-deficient (**C**) after GCRV infection.

Since ITGB1 plays an important role in the process of viral entry into cells, we examined whether the ITGB1b^−/−^ rare minnow can regulate GCRV-induced death compared to the wild-type rare minnow. As shown in Figure 3A, after GCRV infection, the wild-type rare minnow began to die as early as 6 days post-infection (dpi), and all rare minnows died at 8 dpi, with a median survival time of 7 dpi. In contrast, the ITGB1b^−/−^ rare minnow began to die at 8 dpi and continued to 11 dpi following GCRV stimulation. Moreover, the median survival time (9 dpi) of the ITGB1b^−/−^ rare minnow after infection was longer than that of the wild-type rare minnow (7 dpi). Thus, these results showed that the ITGB1b^−/−^ rare minnow exhibited delayed death induced by GCRV infection.

### 2.4. The Efficiency of GCRV Entry Is Reduced in the ITGB1b-Deficient Rare Minnow

**Figure 4:**
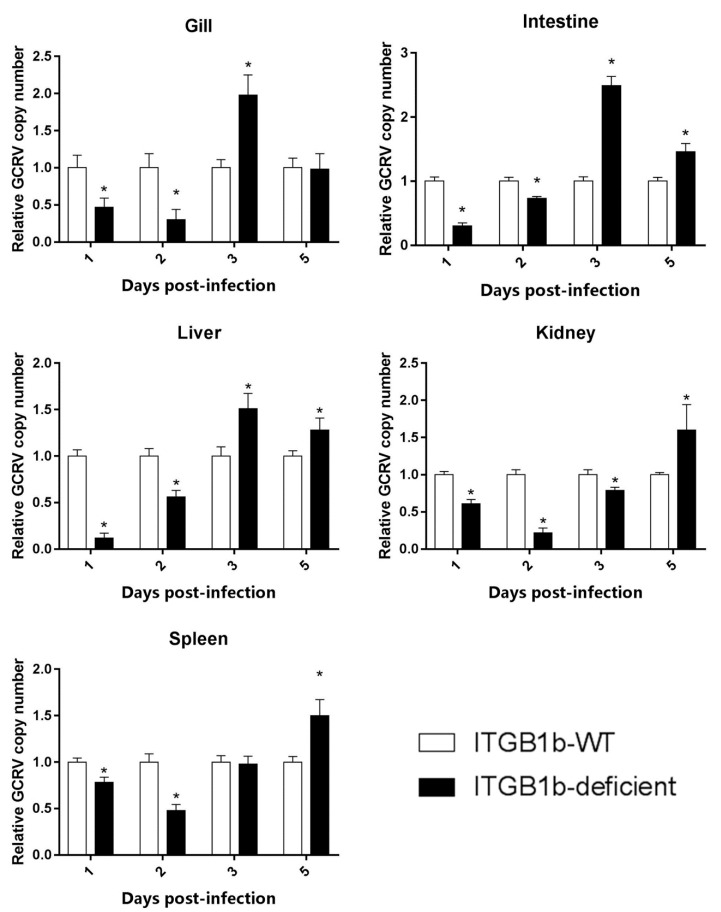
A relative number of GCRV copies in vivo. The relative number of GCRV copies was expressed as the ratio of the level of GCRV S6 segment expression in ITGB1b-deficient fish relative to that of wild-type fish at each time-point. A significant difference (*p* < 0.05, ANOVA) in the number of viral copies between the samples from wild-type and ITGB1b-deficient rare minnows is indicated with an asterisk (*).

To understand the reasons for the prolonged survival time of the ITGB1b^−/−^ rare minnow, the relative GCRV copy number in the gills, intestine, liver, kidney, and spleen was examined. As shown in Figure 4, a significantly (*p* < 0.05, ANOVA) lower viral copy number was observed in ITGB1b^−/−^ rare minnow, compared with wild-type rare minnow at 1 and 2 dpi in all tissues. At 3 dpi, a significantly lower viral copy number was observed in the kidney of the ITGB1b^−/−^ rare minnow in comparison to that of the wild-type minnows. However, in the ITGB1b^−/−^ rare minnow, the viral copy number in the gills, intestine, and liver at 3 dpi, and in the kidney and spleen at 5 dpi, were higher than those in the wild-type minnows. Collectively, these results indicated that the entry efficiency of GCRV in these tissues decreased in the absence of *itgb1b* in the rare minnow.

### 2.5. Clathrin-Mediated Endocytosis Is Attenuated in the ITGB1b-Deficient Rare Minnow

**Figure 5:**
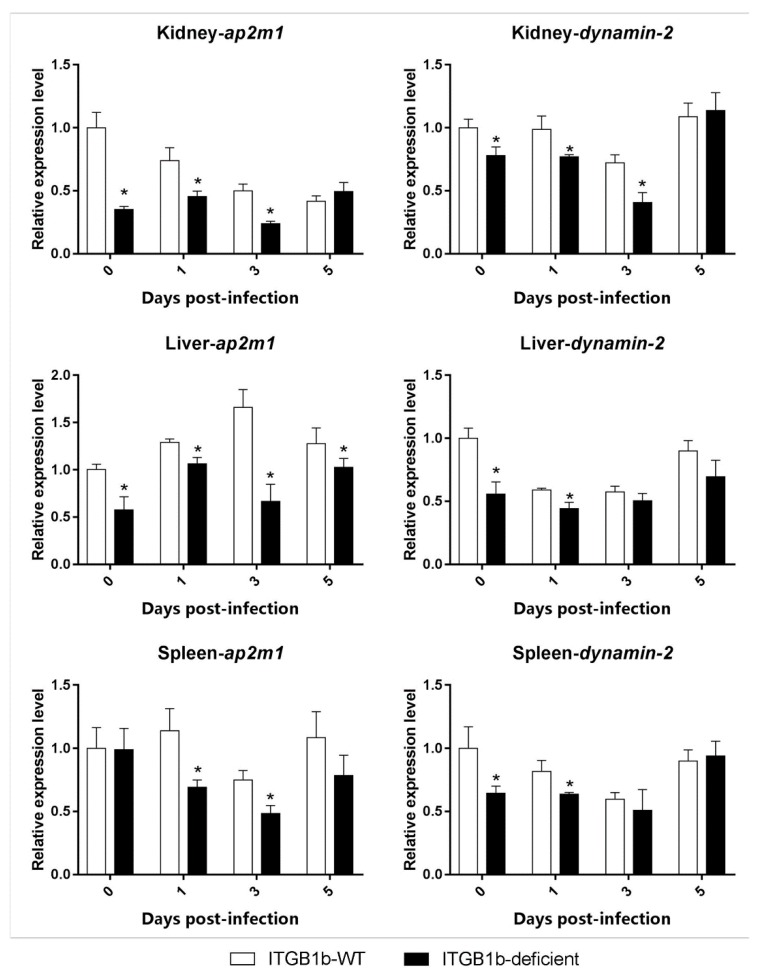
Expression of clathrin-related genes in ITGB1b-deficient and wild-type rare minnows. The relative expression levels were calculated as the ratio of gene expression level in ITGB1b-deficient fish relative to that in wild-type fish at 0 dpi. A significant difference (*p* < 0.05, ANOVA) in gene expression level between the wild-type and ITGB1b-deficient rare minnows is indicated with an asterisk (*).

To investigate the role of *itgb1b* in GCRV entry into cells, the differences in the level of endocytosis-related gene expression (*jam-a*, *ap2m1*, *dynamin-2* and *caveolin-1*) between the ITGB1b-deficient and wild-type rare minnow was compared. As shown in Figure 5, in the kidney, liver, and spleen, the level of *ap2m1* expression in the ITGB1b-deficient minnows was significantly lower at 1 and 3 dpi than the wild-type minnows. Furthermore, in the three tissues, the level of *dynamin-2* expression was significantly lower at 0 and 1 dpi than the wild-type.

**Figure 6:**
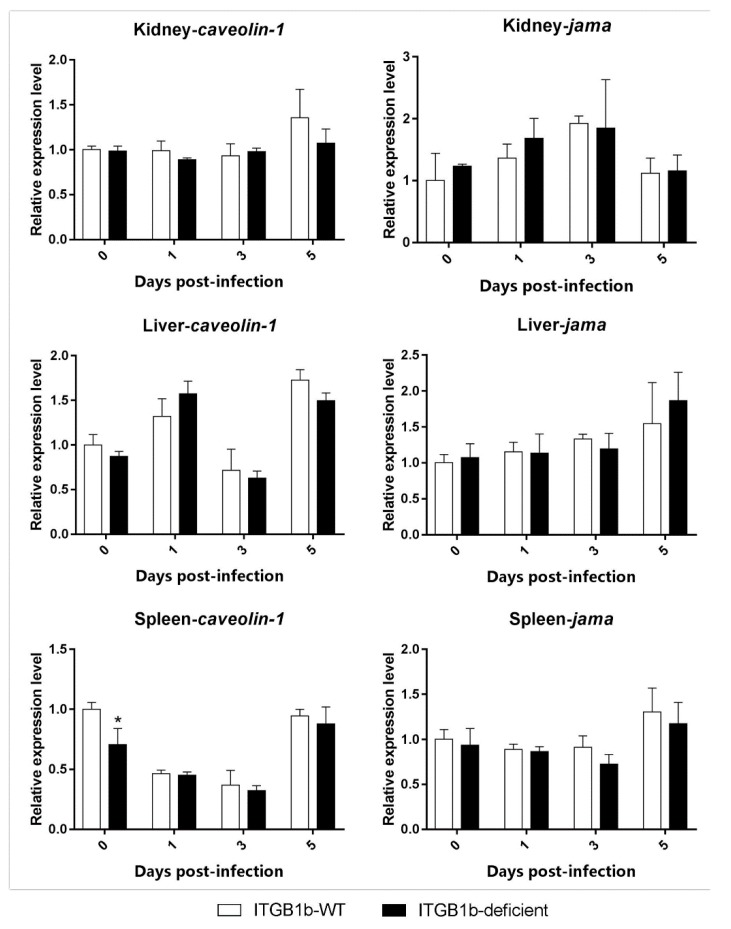
Expression of *jama* and *caveolin-1* genes in ITGB1b-deficient and wild-type rare minnows. The relative expression levels were calculated as the ratio of gene expression level in ITGB1b-deficient fish relative to that in wild-type fish at 0 dpi. A significant difference (*p* < 0.05, ANOVA) in gene expression level between the wild-type and ITGB1b-deficient and wild-type rare minnows is indicated with an asterisk (*).

Moreover, the level of *jam-a* and *caveolin-1* expression during the infection did not significantly differ between the two groups (Figure 6).

### 2.6. GCRV-Induced Apoptosis Is Attenuated in ITGB1b-Deficient Rare Minnows

**Figure 7:**
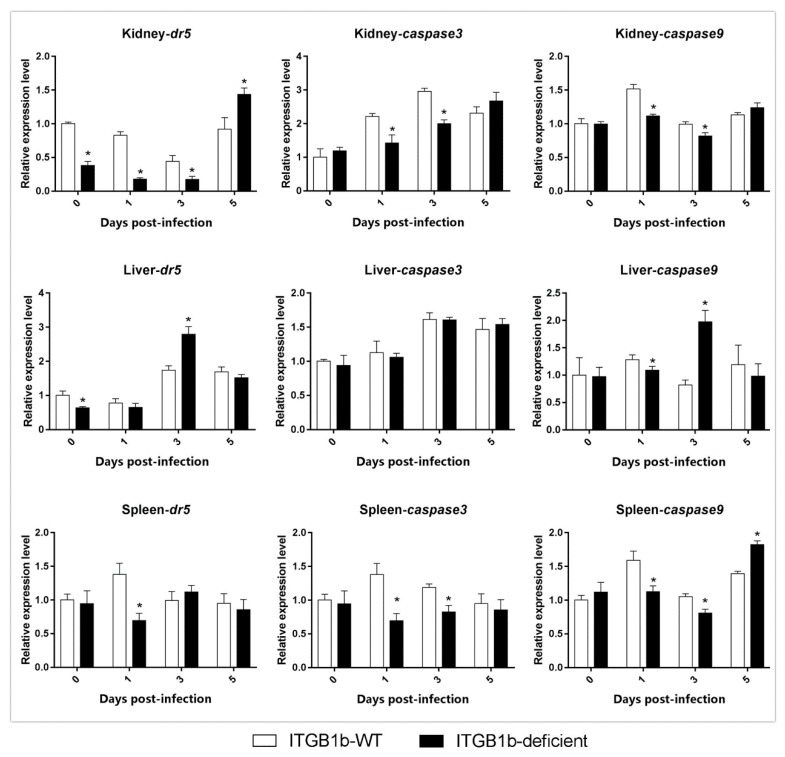
Expression of apoptosis-related genes in ITGB1b-deficient and wild-type rare minnows. The relative expression levels were calculated as the ratio of gene expression level in ITGB1b-deficient fish relative to that in wild-type fish at 0 dpi. A significant difference (*p* < 0.05, ANOVA) in gene expression level between the wild-type and ITGB1b-deficient and wild-type rare minnows is indicated with an asterisk (*).

To investigate the reason for the delayed death of the ITGB1b-deficient rare minnows, the differences in the level of apoptosis-related gene (*dr5*, *caspase-3*, *caspase-9*, *bid*, and *bax*) expression between ITGB1-deficient and wild-type rare minnows were compared. As shown in Figure 7, the level of *caspase-3* and *caspase-9* expression in the kidney and spleen of the ITGB1b-deficient rare minnow were significantly lower compared to the wild-type group at 1 and 3 dpi; however, the level of *dr5* and *caspase-9* expression in the liver of the ITGB1b-deficient rare minnows was significantly higher than the wild-type group at 3 dpi.

**Figure 8:**
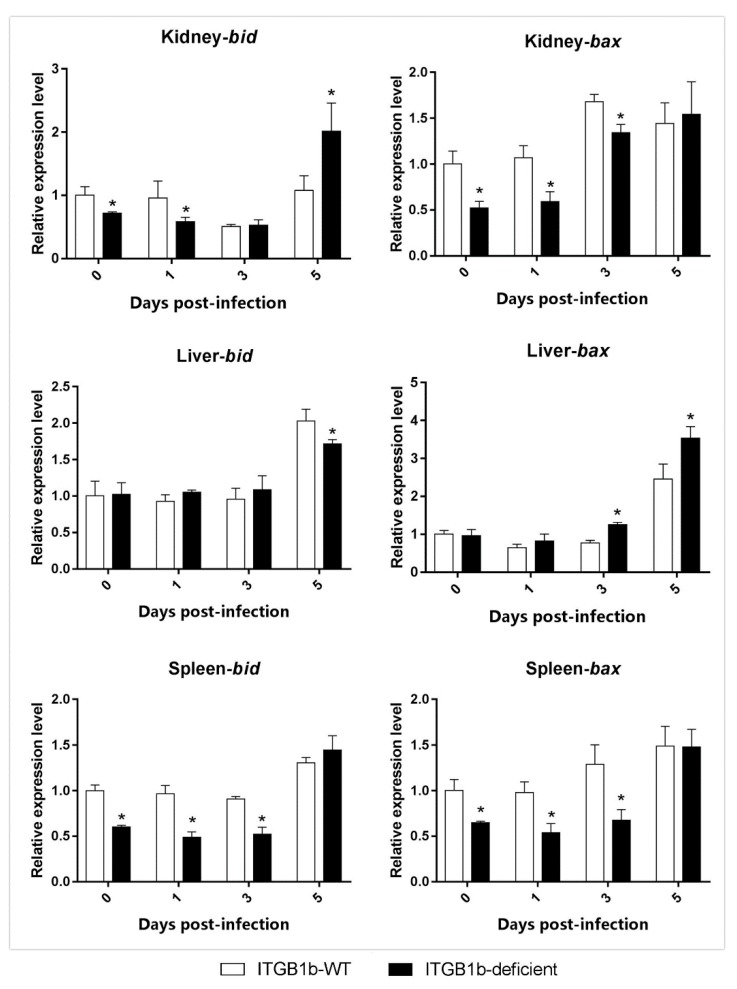
Expression of apoptosis-related genes in ITGB1b-deficient and wild-type rare minnows. The relative expression levels were calculated as the ratio of gene expression level in ITGB1b-deficient fish relative to that in wild-type fish at 0 dpi. A significant difference (*p* < 0.05, ANOVA) in gene expression level between the wild-type and ITGB1b-deficient and wild-type rare minnows is indicated with an asterisk (*).

Moreover, as shown in Figure 8, in the kidney and spleen of the ITGB1b-deficient rare minnow, the level of *bid* expression was significantly lower than that in the wild-type minnows at 0 and 1 dpi, and the level of *bax* expression was observed significantly lower than that of the wild-type minnows at 0, 1, and 3 dpi. In the liver of the ITGB1b-deficient rare minnow, *bax* expression was significantly higher than that of the wild-type minnows at 3 dpi, whilst the expression level of the *bid* was comparable to that of the wild-type at 0, 1, and 3 dpi.

## 3. Discussion

GCRV induces apoptosis in the cells of infected grass carp, where we have been generated a *Bid*-deficient rare minnow to investigate the possible role of the *bid* in GCRV-triggered apoptosis [18]. However, the pathway by which GCRV enters grass carp cells remains unclear. It is important to note that many viruses use integrins as receptors for entry into host cells [19,20,21,22,23,24]. In addition, an increasing number of viruses have been associated with integrins that have different functions beyond viral binding [25]. For example, reovirus internalization is mediated by ITGB1 protein and most likely enters cells via clathrin-dependent endocytosis [26]. Moreover, when bound to integrin αvβ3, dengue virus induces actin cytoskeletal rearrangement [27], whereas herpes simplex virus is transmitted to lipid rafts and dynamin-2-dependent acidic compartments [28]. However, in teleost fish, the role of *itgb1* in GCRV infection remains unclear. In the present study, we mutated the *itgb1b* gene in rare minnows to investigate its potential role by comparing the expression patterns of endocytic and apoptosis-related genes between an ITGB1b-deficient rare minnow and wild-type rare minnows, before and after GCRV infection.

In previous studies, due to its biological characteristics (small, easy to culture, adaptable to a wide temperature range), a relatively short life cycle, and increased vulnerability to GCRV, rare minnow has been used to study GCRV virus infection [29,30]. To elucidate the role of *itgb1b* in the context of GCRV infection, we obtained ITGB1b^−/−^ rare minnows using the CRISPR/Cas9 system. We verified *itgb1b* to be successfully knocked out by RT-PCR, indicating that the technique was feasible and effective for editing rare minnow genes. In the present study, the median survival time and death time of the ITGB1b^−/−^ rare minnows was significantly prolonged following infection with GCRV. The RT-qPCR results revealed that the viral copy numbers in each tissue (gill, intestine, liver, kidney, and spleen) of the ITGB1b^−/−^ rare minnows were decreased, compared to wild-type fish during the early stages of infection (1 and 2 dpi). Other studies have demonstrated that ITGB1-specific antibodies can effectively inhibit the entry of Hepatitis C Virus (HCV) in vitro, confirming that ITGB1 is a cofactor for HCV entry into cells [31]. In addition, experiments with MKF-ITGB1^−/−^ mouse cells showed that the lack of ITGB1 resulted in a reduction in WNV virions by more than 70% to 90%; however, no direct interaction between ITGB1 and the virus was observed [17]. Collectively, these findings indicate that the entry efficiency of GCRV is reduced in ITGB1b^−/−^ rare minnows, and the survival time is prolonged after infection.

One way in which reoviruses enter cells is by first attaching to carbohydrates and JAM-A (junction adhesion molecule-A), which are then internalized by the ITGB1-mediated endocytic pathway [32,33]. The NPXY (Asn-Pro-X-Tyr) motif of ITGB1 interacts with the μ2 subunit of the AP-2 (adaptor protein complex 2) complex, which recruits clathrin and triggers clathrin-mediated endocytosis, for which Dynamin-2 is essential for clathrin-mediated endocytic vesicle formation [34]. A previous study showed that CRISPR/Cas9 effectively knocked out *jam-a* and reduced GCRV infection in grass carp kidney cells [35]. To validate the role of *itgb1b* in clathrin-mediated endocytic GCRV, we compared the expression of *jam-a*, *ap2m1*, *dynamin-2*, and *caveolin-1* between ITGB1b^−/−^ and wild-type rare minnows. The results showed that although the level of *jam-a* and *caveolin-1* expression did not change significantly during the infection, the expression of *ap2m1* and *dynamin-2* in the various tissues exhibited significant changes. The levels of *ap2m1* and *dynamin-2* expression in the kidney, spleen, and liver was significantly lower than those in the wild-type at 0 and 1 dpi. Correspondingly, the number of viral copies in three tissues were also lower than that of the wild-type at the same time. This suggested that in the ITGB1b-deficient rare minnow, the absence of *itgb1b* may reduce the efficiency of clathrin-mediated endocytosis, resulting in reduced levels of infection. However, in the gills, intestines, and liver, there may be other ways in which GCRV does not enter the cell by clathrin-mediated endocytosis via ITGB1. This is likely the case in the liver, where both *ap2m1* and *dynamin-2* in ITGB1b-deficient rare minnows were lower than that of the wild-type minnows early during infection. However, the number of viral copies in the liver was higher than that of the wild-type at 3 dpi, whereas the number of viral copies in the kidney was higher than that of the wild-type until 5 dpi. Previous studies have shown that reovirus infection in *ITGB1*-deficient mice is not prevented, but rather results in low levels of infection [36]. Therefore, reoviruses may have other ways of entering the cell, such as Caveolin-1 mediated endocytosis or through lipid rafts [37]. In addition, although we have not amplified it, *itgb1b* may have a homologous copy in rare minnow, where the other one may also be able to play certain functions. In short, the absence of *itgb1b* may lead to a decrease in the efficiency of clathrin-mediated endocytosis, resulting in a decrease in GCRV entry efficiency.

Studies have shown that reoviruses can induce cell death through death receptor-associated (extrinsic) and mitochondrial (intrinsic) apoptotic pathways [38]. In the extrinsic pathway, reovirus-infected cells up-regulated *dr5* and released TRAIL (TNF-related apoptosis-inducing ligand), leading to the activation of *caspase-3* through a series of regulators [39,40]. In the intrinsic pathway, the release of cytochrome c from mitochondria activates *caspase-9*, which further induces the activation of *caspase-3* [41]. Furthermore, the induction of caspases by GCRV was observed earlier in a grass carp cell line [42]. Our results showed that in the kidney and spleen, the level of *caspase-3* and *caspase-9* expression in the ITGB1b^−/−^ rare minnow was lower than that in the wild-type minnows at 1 dpi and 3 dpi. Moreover, the expression levels of caspase-3 and caspase-9 in the liver were not higher than that of the wild-type minnows at 0 and 1 dpi. Therefore, we hypothesized that during the early stages following infection with GCRV, the absence of *itgb1b* may lead to a decrease in the efficiency of clathrin-mediated endocytosis in the ITGB1b^−/−^ rare minnow, which reduces the number of GCRV copies and attenuates the stimulation of apoptosis.

Moreover, active NF-κB (nuclear factor kappa-light-chain-enhancer of activated B cells) induces the expression of anti-apoptotic genes (e.g., Bcl-2), leading to the inhibition of apoptosis [43], and the virus itself may encode some proteins that inhibit apoptosis to facilitate replication [44]. For example, the virus may encode a viral homolog of Bcl-2 (e.g., Epstein-Barr virus BHRF1 (BamHI fragment H rightward open reading frame 1) protein and the adenovirus E1B 19K protein), which can inhibit pro-apoptotic proteins, such as BAX (Bcl-2 Associated X Protein) and BAK (Bcl-2 homologous antagonist killer) [45]. In our previous studies, we have been generated a *Bid*-deficient rare minnow to investigate the possible role of the *bid* in GCRV-triggered apoptosis, and found that *Bid*-deficient rare minnow attenuated GCRV-induced apoptosis [18]. Our study demonstrated that in the kidney and spleen of the ITGB1b-deficient minnows, the level of the *bid* and *bax* expression was significantly lower than that of the wild-type minnows at 0 and 1 dpi. In contrast, the level of *bid* and *bax* expression in the ITGB1b-deficient minnows was not much different or higher than that of the wild-type minnows in the liver. Although the virus can inhibit apoptosis and allow it to proliferate for long periods, the virus eventually induces its death and then spreads to adjacent cells [46]. Therefore, we hypothesized that in the kidney and spleen of the ITGB1b-deficient rare minnow, the virus has a low number of copies. Thus, for the virus to have enough time to replicate, cell apoptosis is inhibited and is reflected in the delayed death of ITGB1b-deficient rare minnow. In the liver of the ITGB1b-deficient rare minnows, since the number of viral copies is sufficient, apoptosis in the liver may not be inhibited.

## 4. Materials and Methods

### 4.1. Ethics Statement

All animal experiments were conducted according to the Guide for the Care and Use of Laboratory Animals, and the protocol was approved by the Institute of Hydrobiology, Chinese Academy of Sciences. The reference number obtained was Y11201-1-301 (Approval date: 30 May 2016). All surgery was performed under eugenol anaesthesia (100 mg/L) to minimize suffering.

### 4.2. Experimental Animals and Sample Collection

Rare minnows were raised under standard laboratory conditions at the China Zebrafish Resource Center (CZRC). Adult rare minnows were maintained in a standard tank of an automatic fish housing system. Healthy adult female and male fish aged four to five months old were kept in separated tanks and mated once a week to get progeny. Three mature rare minnows were collected, and RNA from the gill, intestine, liver, spleen, kidney, skin, muscle, heart, and brain were prepared for amplification of the *itgb1b* cDNA sequence.

### 4.3. Cloning and Sequence Analysis of ITGB1b

Total RNA was extracted from healthy grass carp and rare minnow tissues using Trizol reagent (Invitrogen, Carlsbad, CA, USA). Total RNA treated with DNase I (Promega, Madison, WI, USA) was used as a template and oligonucleotide (dT)-universal primer was used as a control for reverse transcriptase (TOYOBO, Osaka, Japan), for the first strand of cDNA synthesis. Primers (Table 1) for amplification of *itgb1b* were designed based on the sequences obtained by BLAST analysis sequences of zebrafish *itgb1* with the draft genome of grass carp and rare minnow [rare minnow genome is unpublished data]. Homologous *itgb1* sequences from other species were obtained using the BLAST program (http://blast.ncbi.nlm.nih.gov/Blast.cgi). A phylogenetic tree was constructed based on the full-length amino acid sequences of ITGB1 proteins using MEGA7 software (http://www.megasoftware.net/).

### 4.4. Cas9 Target Site Design and sgRNA Synthesis

The CRISPR/Cas9 system was used to generate *ITGB1b*^−/−^ rare minnows. The Cas9 target site of *itgb1b* was designed using the ZIFIT Targeter (http://zifit.partners.org/zifit/Introduction.aspx) online tool, as described in Reference [47]. A pMD19T-gRNA vector containing a portion of the guide RNA sequence was used for the study, as outlined in Reference [46]. Transcription templates for specific sgRNA (small guide RNA) synthesis were PCR amplified using the primers listed in Table 1. The sgRNA was separately transcribed and purified using T7 RNA polymerase (NEB, Ipswich, MA, USA) and Trizol reagent (Sigma, St. Louis, MO, USA).

### 4.5. Cas9 mRNA Synthesis

The Cas9 nuclease expression vector, pXT7-hCas9, was used to transcribe Cas9 mRNA in vitro [48]. First, XbaI (NEB, Ipswich, MA, USA) linearized the vector. Capped Cas9 mRNA was then synthesized using a mMESSAGE mMACHINE mRNA Transcriptional Synthesis Kit (Ambion, Foster City, CA, USA). Cas9 mRNA was purified using an RNeasy Mini Kit (QIAGEN, Hilden, Germany).

### 4.6. Microinjection and Identification of Mutations

Cas9 mRNA and sgRNA were co-injected into rare minnow embryos during the single-cell stage. Approximately 2 nL of a solution containing 400 ng/μL Cas9 mRNA and 60 ng/μL sgRNA was injected into each rare minnow embryo. Genomic DNA was extracted from normally developing embryos 40 h after injection for the detection of mutations. Specific target sites were amplified using PCR (primers are listed in Table 1) and sequenced by Qingke (Shanghai, China).

### 4.7. Production of ITGB1b-Deficient Rare Minnows

After obtaining sexually mature F0 rare minnows, we crossed the F0 fish with the wild-type minnows. F1 embryos were harvested and sequenced to confirm that these mutations were inherited 40 h after fertilization. Once the desired mutant was identified, sequencing confirmed that the F1 mutant carrier produced F2, producing 25% homozygous wild-type, 50% heterozygous, and 25% homozygous mutant progeny, as explained in Reference [49]. Progeny of the homozygous F2 mutants were selected for further study. To confirm whether the CRISPR/Cas9 system induced a deletion mutation in *itgb1b*, nine tissues were obtained from five *ITGB1b*-deficient and wild-type rare minnows (gill, intestine, liver, spleen, kidney, muscle, heart, brain, and skin). The relative level of *itgb1b* mRNA expression in the different tissues was detected with a CFX96 real-time PCR detection system (Bio-Rad, Hercules, CA, USA) using RT-qPCR. The *β-actin* was used as a reference gene. Specific RT-qPCR primers for *β-actin* and *itgb1b* are listed in Table 1. The cycling procedure for RT-qPCR was as follows: 1 cycle at 95 °C for 2 min, and 15 cycles at 95 °C for 15 s, 58 °C for 15 s, and 72 °C for 30 s, followed by verification of the amplification of a single product by a dissociation curve analysis. All data are expressed as the mean ± standard deviation of three replicates. The level of *itgb1b* expression was calculated using the 2^−ΔΔCT^ method, as described in Reference [50].

### 4.8. GCRV Infection

In the virus infection experiment, 81 individuals of the *ITGB1b*-deficient and 95 wild-type rare minnows (2–3 g body weight) were immersed in 6% saline for 2 min. These fish were then collected and immersed in a solution containing GCRV (50 mL of virus suspension plus 450 mL of culture water: titer: 2.97 × 10^2^ RNA copies/μL) for 20 min. Finally, all fish were transferred to aerated fresh water and incubated at 28 °C.

### 4.9. Detection of the Relative Copy Number of GCRV and Genes

Each day, three individuals were obtained from the *ITGB1b*-deficient and wild-type groups, respectively. The gill, intestine, liver, kidney, and spleen were collected before (0 days) and (1, 3, and 5 days) after GCRV exposure. All samples were used for total RNA extraction and cDNA synthesis, respectively. The relative copy numbers of the virus were examined by RT-qPCR using specific primers for the S6 segments of the GCRV (Table 1). β-actin was introduced as a reference gene (Table 1). The procedure and reaction mixture for RT-qPCR were as described above. Data are expressed as the mean ± standard deviation of three replicates.

### 4.10. Statistical Analysis

The statistical significance between wild-type and ITGB1b-deficient rare minnows was determined by a one-way ANOVA and Fisher’s least significant difference (LSD) posttest. When *p* < 0.05, the difference was considered to be significant.

## 5. Conclusions

In conclusion, *ITGB1b*^−/−^ rare minnows were used in the present study as a model to investigate the possible role of *itgb1b* in GCRV infection and the triggering of apoptosis. After *ITGB1b*^−/−^ rare minnows were infected with GCRV, clathrin-mediated endocytosis-related genes in the liver, spleen, and kidney were significantly down-regulated during the early stages of infection compared with wild-type rare minnows. These findings indicate that clathrin-mediated endocytosis is reduced in *ITGB1b*-deficient rare minnows during infection, resulting in decreased efficiency of GCRV entry into host cells. The difference in the level of apoptosis-related gene expression between *ITGB1b*-deficient and wild-type rare minnows explains the delayed death of the *ITGB1b*^−/−^ rare minnows. These findings provide new insight into GCRV infection, and the subsequent induction of apoptosis. Moreover, understanding the processes of *itgb1b*-mediated GCRV endocytosis contributes to the broader study of viral infection in teleost fish.

## Figures and Tables

**Table 1:** Sequences of primers used in the analysis.

Primers	Sequences (5′to 3′)	Usage
ITGB1b-F	ATATCAGCTCTACTAGGATTTGTCT	*ITGB1B* cDNA cloning
ITGB1b-R	AATGTTTCTCATATTGGGGGTTCAC	
q-ITGB1b-F	GGCTACCTGCTGGTGTGTCC	qRT-PCR for *ITGB1B*
q-ITGB1b-R	TCGTTGAAGCCCAGCGGTTT	
gRNA-F	TGTAATACGACTCACTATAGGAGAAGCAGGG AACACTGGTTTTAGAGCTAGAAATAGC	gRNA amplification
gRNA-R	AAAAAAAGCACCGACTCGGTGCCAC	
T-F	ATGTTCAAGAGGGCAGAGGA	Target site detection
T-R	ACTGGAGACTTTGCCCGTAG	
S6-F	AGCGCAGCAGGCAATTACTATCT	qRT-PCR for GCRV segment S6
S6-R	ATCTGCTGGTAATGCGGAACG	
β-F	TGTAGCCACGCTCGGTCAG	qRT-PCR for β-actin
β-R	GGTATCGTGATGGACTCTGGTG	
AP2-F	TGCGTTCCGTGTGAACGTGA	qRT-PCR for AP2m1
AP2-R	TGGTAACGGCAGCCAACCAG	
Dynamin2-F	GCACCATATCCACCCCCGTG	qRT-PCR for Dynamin-2
Dynamin2-R	TTGGACCTCTCACTGCGGGA	
JAMA-F	TGGTGAAGGCGTCACTCAGC	qRT-PCR for JAM-A
JAMA-R	TGTCTCCGTGCTCAAGGTGC	
Caveolin-F	GCCATGAGAGGCTGCAGGAG	qRT-PCR for Caveolin-1
Caveolin-R	ACAGAGGAAGCCGTGTGGGA	
DR5-F	TCCTCTCCGCTCCAGCCATT	qRT-PCR for DR5
DR5-R	AAGAGTTGGCGGTGTTGGGG	
Caspase-9F	CGTCCGTCTGGTCATCTATCC	qRT-PCR for Caspase-9
Caspase-9R	GAACTGAGGCAAACCACAATC	
Caspase-3F	TCGTAATGGGACAGACAGGG	qRT-PCR for Caspase-3
Caspase-3R	GCCATCGGTGCCATAAATC	
Bid-F	ACAGAAACGTCAACGTTCCTCA	qRT-PCR for Bid
Bid-R	CTACCTGAGCCTCTACAGCATTGA	
Bax-F	TCTGTGCGTCCGCCATCTTC	qRT-PCR for BAX
Bax-R	GCTCAGCCTCAGTTACGCCC	

## Abbreviations

GCRV	grass carp reovirus
ITGB1	integrin β-1
Dpi	days post-infection
dsRNA	double-stranded RNA
sgRNA	small guide RNA
OCSS	oral squamous cell carcinoma
SPC-A-1	lung cancer cell line
WT	wild-type
JAM-A	junction adhesion molecule-A
AP-2	adaptor protein complex 2
NPXY	Asn-Pro-X-Tyr
NF-κB	nuclear factor kappa-light-chain-enhancer of activated B cells
BAX	Bcl-2 Associated X Protein
BAK	Bcl-2 homologous antagonist killer
DR5	death receptor 5
BHRF1	BamHI fragment H rightward open reading frame 1
HCV	hepatitis C virus
RT-qPCR	real-time quantitative PCR
CZRC	China Zebrafish Resource Center
TRAIL	TNF-related apoptosis-inducing ligand
Ct value	cycle threshold value
ANOVA	analysis of Variance
